# Propofol in the Pediatric Intensive Care Unit, a Safe and Effective Agent in Reducing Pain and Sedation Infusions: A Single-Center Retrospective Study

**DOI:** 10.7759/cureus.27925

**Published:** 2022-08-12

**Authors:** Sruti Uppuluri, Enrique G Villarreal, Vincent Dorsey, Faeeq Yousaf, Juan S Farias, Saul Flores, Rohit S Loomba

**Affiliations:** 1 Pediatrics, Advocate Children's Hospital, Oak Lawn, USA; 2 Pediatrics, Instituto Tecnológico y de Estudios Superiores de Monterrey (ITESM), Monterrey, MEX; 3 Pharmacology, Advocate Children's Hospital, Oak Lawn, USA; 4 Pediatrics, Children's Mercy Hospital, Kansas City, USA; 5 Cardiology, Texas Children's Hospital, Houston, USA; 6 Cardiology, Advocate Children's Hospital, Oak Lawn, USA

**Keywords:** pediatric anesthesiology, deep sedation, pain management, pediatric intensive care units, propofol

## Abstract

Introduction

Propofol has long been used as an anesthetic agent during pediatric surgery. Its use in pediatric intensive care units has been largely controversial. A beneficial use of propofol is to facilitate weaning of other pain and sedation infusions such as opiates and benzodiazepines. However, some have advocated to not use propofol due to fear of possible adverse effects including propofol infusion syndrome and hemodynamic instability. The purpose of this study was to determine both the safety of propofol infusions in critically ill pediatric patients, as well as the change in the requirement of other pain and sedation infusions by use of a propofol infusion.

Methods

Single-center, retrospective data (January 2011 to January 2020) was obtained manually using a study-specific data extraction tool created for electronic medical records. The data obtained included variables of interest that measured physiological parameters and pain/sedation infusion (morphine, fentanyl, hydromorphone, midazolam, and dexmedetomidine) rates during three time periods: before propofol initiation, immediately after discontinuation, and four hours after discontinuation. The physiological parameters were then compared to the pain and sedation infusion rates using paired Wilcoxon signed-rank tests.

Results

There was a total of 33 patients with an average age of 11.1 years who were given a median initial propofol infusion of 50 mcg/kg/min with a peak dose of 75 mcg/kg/min over an average of eight hours. Age had a weak and insignificant correlation with initial rate and duration and a moderate and significant correlation with peak rate and duration. Physiological parameters did not vary at any time point measured. There was a significant reduction in other pain and sedation infusions after discontinuation of propofol.

Conclusion

Propofol infusions are hemodynamically tolerated and the majority of patients who are on other pain and sedation infusions tolerate complete discontinuation of these infusions following propofol discontinuation.

## Introduction

Propofol has long been used as an anesthetic agent during pediatric cardiac surgery. Its use in pediatric intensive care units (PICUs), however, has been largely controversial. Some have advocated that propofol not be used due to fear of possible adverse effects including propofol infusion syndrome and hemodynamic instability. Propofol infusion syndrome manifests as refractory bradycardia and refractory lactic acidosis and is thought to be related to cumulative propofol dose [1,2]. While propofol infusion syndrome has been reported and is associated with significant morbidity and mortality, the actual prevalence of propofol infusion syndrome in pediatric patients is relatively low at approximately 1-4%, particularly considering its ubiquitous use in the perioperative setting [3,4].

A beneficial use of propofol may be to facilitate weaning of other pain and sedation infusions such as opiates and benzodiazepines [5]. Anecdotally, many share the efficacy of so-called “propofol washouts” to help wean other pain and sedation infusions; however, there is little objective data demonstrating this. Propofol may mediate this “washout” effect by increasing the expression of µ-opioid receptors, allowing for a lesser requirement of opiates [6]. Additionally, when compared to other anesthetics, propofol may diminish the length of withdrawal symptoms and promote early extubation in patients with large opiate requirements [7].

There is scarce data on the safety and utility of propofol infusions in the pediatric intensive care setting [8]. Given that propofol usage may be beneficial in this setting, further investigation is warranted. This study’s objectives were two-fold: the first was to characterize the safety of propofol infusions in patients cared for in the PICU or pediatric cardiac intensive care unit (PCICU) and the second was to determine the change in the requirement of other pain and sedation infusions with a propofol infusion.

## Materials and methods

Patient identification

Patients cared for in the PICU or PCICU of the Advocate Children's Hospital, Oak Lawn, Illinois, United States, who were placed on a propofol infusion between January 1, 2011, and January 1, 2020, were retrospectively identified using electronic medical records. Patients must have been on a continuous infusion of propofol that lasted for a minimum of an hour and have received the propofol infusion in the PICU or the PCICU. Patients in the operating room were excluded from this study. As some young adults with childhood illnesses were cared for in these two units as well, we decided to include these patients as well. The Institutional Review Board of the Advocate Children's Hospital approved this study (approval number: 1943679-1). This study complies with the Helsinki Declaration.

Variables of interest

The following variables of interest were identified prior to the study: heart rate, systolic blood pressure, diastolic blood pressure, serum lactate, FLACC (face, legs, activity, cry, consolability) scale, need for vasoactive support as vasoactive-inotropic score, and pain and sedation infusion doses.

The pain and sedation infusions included in this study were morphine, fentanyl, hydromorphone, midazolam, and dexmedetomidine. Infusion rates were expressed in mg/kg/hr. A composite was created for all opioids by converting the opioid doses to morphine equivalents. A conversion factor of 7 was used for hydromorphone while a conversion factor of 0.1 was used for fentanyl [9].

Data extraction

Data were extracted manually from the electronic medical records using an electronic data collection tool that was created specifically for this study. The composite opioid dose was calculated in morphine equivalents as described above while the vasoactive-inotropic score was calculated as previously described elsewhere [10].

Physiologic, vasoactive, and pain/sedation infusion data was collected for the three defined timepoints: immediately before the initiation of propofol infusion, immediately after discontinuation of propofol infusion, and then four hours after the propofol infusion was discontinued. Serum lactate was only collected prior to the initiation of the propofol infusion and four hours after the propofol infusion was discontinued since serum lactates were not obtained frequently enough to capture for the remaining timepoint.

If a single patient received multiple infusions, only the first one meeting the inclusion criteria was included. If an infusion was restarted before four hours of previous propofol infusion discontinuation then this was treated as a single infusion.

Statistical analyses

The skewness of data was evaluated to determine whether the data were normally distributed or not. Categorical data are presented as absolute frequency and percentage while continuous data are presented as median and range. Spearman's correlation tests were run to assess the correlation between age at the time of the propofol infusion and starting propofol infusion rate, peak propofol infusion rate, and duration of propofol infusion.

Next, physiologic and pain and sedation infusion data were compared between the timepoints using paired Wilcoxon signed-rank tests. The paired Wilcoxon signed-rank test can only compare two related values and thus for a single variable of interest there were three separate paired Wilcoxon signed-rank tests conducted: 1) immediately before the initiation of the propofol infusion versus immediately after the propofol infusion was discontinued, 2) immediately after the propofol infusion was discontinued versus four hours after the propofol infusion was discontinued, and 3) immediately before the initiation of the propofol infusion versus four hours after the propofol infusion was discontinued.

This was done rather than using Kruskal-Wallis one-way analysis of variance analyses to allow for comparison of the values between each timepoint to identify precisely between which timepoints significant differences existed. Using an omnibus test such as the Kruskal-Wallis one-way analysis of variance in which data for all three timepoints for the same variable of interest were compared at once would only demonstrate if a statistically significant difference existed between any two points but not identify specifically between what points the difference existed.

All statistical analyses were done using the user-coded interface of IBM SPSS Statistics for Windows, Version 26.0 (Released 2019; IBM Corp., Armonk, New York, United States). A p-value of less than 0.05 was considered statistically significant for these analyses. Any use of the word “significant” in the text implies statistical significance unless explicitly stated otherwise.

Informal social media survey

An informal two-question survey was conducted on Twitter (Twitter, Inc., San Francisco, California, United States), aimed at those caring for critically ill children. The first question was “Do you think propofol infusions are generally a safe option for sedation in the PICU or the PCICU?”. Response options were “yes” and “no”. The second question was “Do you think propofol infusions can be used to decrease the requirement of other sedative agents in children when used in the setting of a so-called ‘propofol washout’?”. Response options were “yes” and “no”. The survey was left open for 48 hours.

## Results

Cohort characteristics

A total of 33 patients were included in the final analyses. Of these, seven (21.2%) had propofol infusions started in the PCICU while the remainder were in the PICU. The median age was 11.1 years with a range of 0.3-27.1 years. As mentioned in the Methods and Materials section, the decision to include young adults cared for in these units was made *a priori*. Three such patients were included, aged 21, 22, and 27 years. Of the 33 patients included, eight (24.2%) had congenital heart disease (Table 1).

**Table 1:** Cohort characteristics and propofol dosing/duration

Characteristics	Frequency (%) or median (range)
Age (years)	11.1 (0.3 to 27.1)
Congenital heart disease	8 (24.2%)
Need for any additional sedation infusion at any point	22 (66.7%)
Need for fluid bolus during propofol infusion	9 (27.3%)
Vasoactive support before initiation	3 (9.1%)
Vasoactive support at discontinuation	4 (12.1%)
Initial propofol infusion dose (mcg/kg/min)	50 (15 to 100)
Peak propofol infusion dose (mcg/kg/min)	75 (15 to 200)
Duration of propofol infusion (hours)	8 (1 to 222)

The primary reason for admission is outlined in Table 2. The most common reason for admission in patients who had a propofol infusion was ingestion, which was noted in eight (24.2%). The median initial propofol infusion rate was 50 mcg/kg/min. Initial rate ranged from 15 mcg/kg/min to 100 mcg/kg/min. The median peak propofol infusion rate was 75 mcg/kg/min. This ranged from 15 to 200 mcg/kg/min. The average propofol infusion duration was eight hours, with a range of 1 to 222 hours. There were 22 (66.7%) patients who had additional sedation drips at any point during the propofol infusion. Nine (27.3%) of patients required a fluid bolus during the propofol infusion due to inadequate blood flow, hypovolemia, or hypotension. 

**Table 2:** Primary reasons for admission

Reason for admission	Frequency (%)
Airway anomaly	1 (3.0%)
Arrhythmia	2 (6.1%)
Aspiration	1 (3.0%)
Cardiac surgery	5 (15.2%)
Cardiomyopathy	1 (3.0%)
Cardiorespiratory arrest	4 (12.1%)
Cystic fibrosis	1 (3.0%)
Gastrointestinal bleed	1 (3.0%)
Ingestion	8 (24.2%)
Malignancy	3 (9.1%)
Seizures	3 (9.1%)
Traumatic Brain Injury	3 (9.1%)

Correlation of age to propofol infusion characteristics

Age at infusion had a weak and insignificant correlation with starting propofol rate (correlation coefficient -0.02, p=0.89). Age at infusion had a moderate and significant correlation with peak propofol rate (correlation coefficient -0.51, p<0.01). Age at infusion had a weak and insignificant correlation with propofol infusion duration (correlation coefficient 0.01, p=0.95).

Physiologic parameters

There were no significant changes in heart rate, systolic blood pressure, or diastolic blood pressure between any of the three timepoints. The vasoactive-inotropic score was also similar at all three timepoints. Serum lactate was not significantly different when compared before the initiation of the propofol infusion and four hours after the propofol infusion was discontinued (Table 3).

**Table 3:** Impact of propofol on heart rate, blood pressure, vasoactive-inotropic score, lactate levels, and FLACC scale Data presented in median (range) ‡ No significant difference was noted in any of the parameters measured after propofol discontinuation (immediately or after four hours) when compared to the time when propofol was initiated FLACC: face, legs, activity, cry, consolability

Parameters	Prior to the initiation of propofol	Immediately after propofol infusion was discontinued^‡^	Four hours after propofol infusion was discontinued^‡^
Heart rate	103 (60 to 185)	94 (55 to 156)	96 (45 to 168)
Systolic blood pressure	113 (77 to 144)	111 (67 to 160)	108 (66 to 143)
Diastolic blood pressure	69 (44 to 110)	66 (42 to 107)	66 (37 to 115)
Vasoactive-inotropic score	0 (0 to 12.4)	0 (0 to 20)	0 (0 to 15)
Lactate	1.9 (0.4 to 3.4)	--	0.8 (0.6 to 6.4)
FLACC scale	0 (0 to 6)	0 (0 to 5)	0 (0 to 5)

Pain and sedation data

There was a significant reduction in opioid, dexmedetomidine, and midazolam infusion rates immediately after propofol discontinuation as well as four hours after propofol infusion completion when compared to the dose of these infusions prior to propofol initiation. A significant difference in infusion rates at propofol discontinuation and four hours after completion of propofol was only noted for dexmedetomidine (Table 4).

**Table 4:** Dosing changes on other pain and sedation infusions after discontinuation of propofol Data presented in median (range) † Significant difference when compared to before propofol infusion ‡ Significant difference when compared to the time when propofol infusion discontinued

Other pain and sedation infusions	Before initiation of propofol	Immediately after propofol infusion was discontinued	Four hours after propofol infusion was discontinued
Morphine equivalents (mg/kg/hr)	0.12 (0.00 to 5.80)	0.00 (0.00 to 0.17)^†^	0.00 (0.00 to 0.20)^†^
Dexmedetomidine (mcg/kg/hr)	0.70 (0.00 to 2.00)	0.50 (0.00 to 1.80)^†^	0.15 (0.00 to 1.70)^†‡^
Midazolam (mg/kg/hr)	0.06 (0.04 to 0.10)	0.00 (0.00 to 0.04)^†^	0.00 (0.00 to 0.04)^†^

The median infusion rate of opioid and midazolam infusions was zero immediately after the propofol infusion was discontinued and, similarly, four hours after the propofol infusion was discontinued. No patients had to be restarted on an opioid or benzodiazepine infusion during the remainder of the admission. The FLACC scale did not significantly change between the time points.

Informal social media survey

A total of 98 responses were obtained for the first question regarding the safety of propofol infusions for sedation. Of the 98, 63% responded “yes”. A total of 54 responses were obtained for the second question asking about the presumed efficacy of propofol infusions for “washouts”. Of the 54, 87% responded “yes”.

## Discussion

This study describes the use of continuous propofol infusions in the PICU and PCICU settings, adding to the limited existing data in pediatrics. The data from this study demonstrate that propofol infusions are safe in pediatrics. There was no occurrence of propofol infusion syndrome even with infusion durations greater than 100 hours and serum lactate did not differ before and after the propofol infusion. Propofol infusions were hemodynamically tolerated and while fluid boluses were needed in some patients, the vasoactive-inotropic score did not significantly differ before and after the propofol infusion.

More importantly, propofol infusions were shown to decrease infusion rates of other pain and sedation infusions. Twenty-two patients were on other pain and sedation infusions (dexmedetomidine, opioids, or benzodiazepines) when the propofol infusion was started. Nearly all of these patients reduced the other pain and sedation infusions after the initiation of propofol. In our study, the decreased need for pain/sedation infusions was sustained in the four-hour follow-up time.

While “propofol washouts” have been anecdotally described, there is scarce data in the pediatric population to support this practice [5,11-15]. The majority of existing pediatric data on propofol is in the perioperative setting. Propofol has shown to have multiple mechanisms of action including GABA-A receptor agonist, GABA-A receptor positive allosteric modulation, NMDA-receptor agonist, endocannabinoid agonist, and sodium channel antagonist [16,17]. Additionally, propofol has effects on the µ-opioid receptors by upregulating the expression of these receptors and enhancing the activity of the endogenous µ-opioid system [11].

Propofol is a short-acting medication, with a duration of action of 3-10 minutes and a terminal half-life ranging from 1-11 hours [18]. This allows for easy, rapid titration of propofol in the critical care setting to achieve a desired level of sedation. Once an appropriate level of sedation is achieved with propofol, rapid and significant weans in opioid and benzodiazepine infusions can be achieved. The decreased opioid and benzodiazepine requirements remain long after the propofol infusion is discontinued.

Historically, propofol infusions have been avoided in the PICU and PCICU due to the possibility of developing propofol-related infusion syndrome. Diagnosis of propofol-related infusion syndrome is based on the presence of refractory bradycardia with one of the following: 1) hepatomegaly or fatty liver, 2) lipemic plasma, 3) metabolic acidosis, or 4) skeletal muscle breakdown [19]. Propofol-related infusion syndrome has been described to be relatively infrequent, occurring in only 1-4% of children on propofol infusions. The likelihood of propofol infusion syndrome seems to be positively correlated with infusion duration and infusion rate of propofol. In this study, no patients demonstrated findings consistent with propofol infusion syndrome, even those on prolonged propofol infusions [1]. Additional side effects of propofol include suppression of the respiratory drive. Respiratory rate and tidal volume may both decrease with propofol, leading to a decrease in minute ventilation. Decreases in cardiac output have also been demonstrated [20-23].

Findings from this study demonstrate that propofol infusions are hemodynamically tolerated and are associated with a low prevalence of propofol-related infusion syndrome. Its ability to facilitate weans in other pain and sedation infusions makes it a valuable tool for pediatric intensivists. Propofol infusions may be used in children with significant, escalating pain and sedation infusion requirements or in those who are unable to achieve and maintain adequate sedation. Such infusions should be administered with close monitoring. While significant hemodynamic changes appear to be infrequent, appropriate monitoring is necessary for the safe administration of propofol. Changes in systemic oxygen delivery and minute ventilation may be detected by using end-tidal carbon dioxide monitoring, blood gas analysis, and near-infrared spectroscopy.

The informal social media survey was able to lend some insight into what general attitudes regarding propofol infusions are. Over half of the responders felt that propofol infusions are safe when used for sedation in pediatrics. A large majority felt that propofol infusions can be successfully used for “washouts” to reduce the requirements of other pain and sedation infusions.

While this study is novel and clinically pertinent, it is not without its limitations. Firstly, the sample size is small. However, the utilization of paired statistical analyses does help overcome this to a certain degree as paired tests are more adequately powered than independent samples tests with the same sample size. Additionally, this is a single-center retrospective study, which may limit its generalizability to some extent. The role of the informal social media survey here is to simply demonstrate easily assessed sentiment regarding propofol. This is not meant to be a fully validated survey.

This study highlights that pediatric intensivists acknowledge that propofol infusions may be helpful and appear to be generally safe. Anecdotal fears related to propofol and its infrequent adverse effects should be tempered by objective data, with the growing clinical experience being used to identify patient populations in whom propofol may be particularly helpful.

## Conclusions

Propofol infusions are hemodynamically tolerated and have a low prevalence of propofol infusion syndrome in pediatrics. In this retrospective study of 33 pediatric patients receiving propofol in the PICU or PCICU, there were no cases of propofol infusion syndrome. For those who were on other pain/sedation infusions when initiating propofol, the majority tolerated complete discontinuation of these infusions after completion of the propofol infusion.
